# Is cavitation a truly sensible choice for intensifying photocatalytic oxidation processes? – Implications on phenol degradation using ZnO photocatalysts

**DOI:** 10.1016/j.ultsonch.2023.106548

**Published:** 2023-08-03

**Authors:** Varaha P. Sarvothaman, Vijay K. Velisoju, Janardhanraj Subburaj, Mebin S. Panithasan, Shekhar R. Kulkarni, Pedro Castaño, James Turner, Paolo Guida, William L. Roberts, Sanjay Nagarajan

**Affiliations:** a Clean Combustion Research Center (CCRC), King Abdullah University of Science and Technology (KAUST), Thuwal 23955-6900, Saudi Arabia; b Multiscale Reaction Engineering (MuRE) Group, KAUST Catalysis Center (KCC), King Abdullah University of Science and Technology (KAUST), Thuwal 23955-6900, Saudi Arabia; c Department of Chemical Engineering, University of Bath, Claverton Down, Bath BA2 7AY, UK; d Centre for Sustainable Energy Systems, University of Bath, Claverton Down, Bath BA2 7AY, UK

**Keywords:** Wastewater treatment, Phenol, Cavitation, Photocatalysis, Peroxide, Hybrid processes

## Abstract

Phenols are recalcitrant compounds that constitute the majority of organic contaminants in industrial wastewaters. Their removal at large scales require a combination of various processes to reach the desired discharge quality. An extensive body of work has already been published in the area of phenol removal from wastewater, however none of them have focussed on a truly ‘sensible’ approach for coupling advanced oxidation processes (AOPs). Rather, a higher removal efficiency was targeted by unduly complicating the process by combining multiple AOPs. The most influential AOP as the primary method typically driven by the nature of the pollutant should form the basis for a hybrid AOP followed by a complementary AOP to intensify the oxidation process. This strategy is lacking in current literature. We address this knowledge gap directly by systematically identifying the best hybrid process for ZnO mediated photocatalysis of phenol. Either a cavitation mediated pre-treatment of ZnO or cavitation-photocatalysis-peroxide based hybrid AOP was investigated. While the pre-treatment approach led to >25% increase in phenol oxidation compared to bare ZnO photocatalysis, the hydrodynamic cavitation-photocatalysis-peroxide based system was found to have a cavitational yield 5 times higher than its acoustic cavitation counterpart. A new phenomenon known as the ‘*pseudo staggered effect*’ was also observed and established in hydrodynamic cavitation mediated photocatalysis-peroxide hybrid process for the first time. While we demonstrated that cavitation is a truly ‘sensible’ choice to enhance photocatalysis, the nature of the pollutant under investigation must always be the key driver when designing such hybrid AOPs.

## Highlights

• Pre-treatment of photocatalyst using sonication is effective in acidic/alkali media.
• Staggered peroxide dosing instead of one step peroxide addition was investigated.
• Staggered peroxide dosing did not impact cavitation based hybrid AOPs.
• Staggered peroxide dosing had significant impact with only photocatalysis.
• Hybrid AOPs would be sensible when driven by nature of pollutant.

## Graphical abstract

**Figure:**
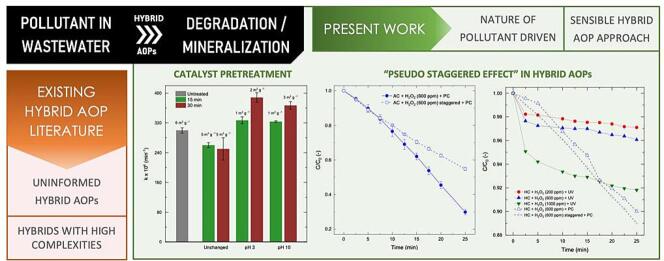

## Nomenclature

**Symbols**
C_0_: initial phenol concentration (ppm)
C_cat_: catalyst loading (g/L)
k: phenol oxidation first order rate constant (min^−1^)
V: liquid volume (mL)

**Abbreviations**
AOP: advanced oxidation processes
AC: acoustic cavitation
HC: hydrodynamic cavitation
PC: photocatalysis
BET: Brunauer–Emmet–Teller
XPS: x-ray photoelectron spectroscopy
ppm: parts per million (mg/L)

**Greek letter**
η: enhancement factor (-)

## 1 Introduction

Oilfield wastewater, known as produced water (PW), is an inevitable by-product of crude oil extraction and refining. Phenol-based recalcitrant pollutants are predominantly found in PW and their removal from wastewater using physical, thermal, chemical, and biological methods have been explored previously [1]. However, these methods suffer from various disadvantages such as not meeting regulatory standards, requiring periodic cleaning, high energy requirement and generation of sludge [2], [3], [4]. To overcome these limitations, advanced oxidation processes (AOP) (for example photocatalysis, Fenton-based processes, O_3_, UV-peroxide, wet air oxidation and cavitation) that can generate in-situ oxidants have been investigated for phenol degradation [5], [6]. Amongst these AOPs, photocatalysis is a promising method that has been explored extensively, especially for phenol removal from wastewater.

In photocatalysis, a semiconductor material is illuminated by light of energy equivalent or higher than its bandgap energy leading to excitation of electrons from its valence to conduction band leaving behind a positive hole, leading to direct or indirect oxidation (via reactive oxygen species) reactions [7]. The reactions predominantly occur on the catalyst surface as opposed to the bulk liquid [8]. It is therefore critical that mass transfer limitations (often a problem for scaling up photocatalytic processes) are overcome to enable photocatalysis for wastewater treatment [9]. Literature focuses on various reactor designs to overcome these drawbacks but are limited to laboratory scale demonstrations and very scarcely at pilot scale. Photocatalytic oxidation has been extensively studied for environmental remediation, particularly for enhancing oxidation rates. However, the presence of oxidation intermediates and changes in solution pH can lead to decreased treatment efficiencies. This may result from competitive inhibition for adsorption on the catalyst surface or alterations in the photocatalyst surface properties over time. Therefore, researchers have explored hybrid processes, such as combining cavitation, peroxide, or ozone with photocatalysis, to improve pollutant removal [10]. However, the inclusion of multiple methods can complicate the process. A systematic integration of AOPs is necessary to understand the true nature of the process for ‘sensible’ hybrid process development.

Cavitation is the most used method with photocatalysis to enable increased pollutant oxidation rates. Previous reports present synergistic indices to compare and explain the efficiencies of hybrid AOPs, overall this results in a substantial enhancement in the rates of the pollutant destruction [11]. The efficacy of sonophotocatalysis is dependent on the mode of operation, capacity of the reactor, operating pH, treatment time, catalyst and oxidizing agents used and aeration [12], [13]. Degradation of various pollutants by coupling acoustic cavitation and photocatalysis such as 2-chlorophenol [14], Acid Orange 52 dye [15], perfluorooctanoic acid [16] and Bisphenol A [17] have been reported in the literature. Hydrodynamic cavitation (HC) has also been coupled with photocatalysis (PC), for the degradation of dyes and phenolics [18], [19]. The coupling of photocatalysis with HC [20] is more recent than the coupling of photocatalysis and acoustic cavitation [21].

Existing instances always added photocatalysis to an optimized cavitation process for enhanced oxidation rates, but not vice versa. However, this approach is not suitable or universally applicable for all pollutant types. While cavitation can result in generation of local hot spots, formation of highly reactive free radicals, increased surface area of catalysts (resulting from fragmentation and potential deagglomeration [21], [22]) and enhancement in the mass transfer [11], the nature of the pollutant plays a significant role in determining the reaction rates. The nature of the pollutant determines its location in the bulk liquid during cavitation, the locations being: inside the bubble, bubble–liquid interface or the bulk liquid medium [23]. For instance, hydrophobic organic pollutants have been successfully degraded (pyrolyzed) as they can migrate to the bubble core [24]. Hydrophilic pollutants like phenol degrade by relying on radicals diffusing to the bulk medium or the bubble–liquid interface for oxidation. Hence, cavitation as a standalone process could not enable degradation of phenol [25]. Coupling cavitation and photocatalysis should therefore be performed by first understanding the degradation of phenol via photocatalysis followed by coupling cavitation and not vice versa.

The degradation of phenol and substituted phenols by AOPs has been studied quite extensively [26], [27], [28]. Especially, heterogeneous photocatalysis [29] has gained wide interest due to the rapid breakdown of organic pollutants. For the phenol degradation pathway by photocatalysis, several intermediates have been reported [30], [31].The intermediates produced during the process can hinder the complete oxidation of phenol due to competitive surface adsorption. Therefore, higher initial oxidation rates are observed compared to the overall process oxidation rates. Hence, maximising photocatalytic oxidation performance by coupling with cavitation is imperative for surface cleansing effects and improved mass transfer. Table 1 presents the use of cavitation-based processes and the incorporation of additional AOPs to supplement oxidants and accelerate the degradation process.

**Table 1:** Summary of literature investigating hybrid advanced oxidation processes for pollutant degradation.

Pollutant (s)	Oxidation processes investigated	Key findings	Reference
5-fluorouracil and lovastatin drugs	AC, Copper nanoparticle and H_2_O_2_	>90% degradation efficiency reported for optimal combination of AC, Copper, and H_2_O_2_	Dinesh et al. [32]
Methylene blue	HC, Bi-doped TiO_2_ based PC and H_2_O_2_	∼ 95% dye decolorization over 10-L operating volume, using visible light PC	Kumar et al. [33]
Azorubine	AC, Fe^2+^ ions, and UVC irradiation for persulfate activation	Extensive interaction of parameters performed. UVC irradiation most effective among the three techniques for persulfate activation	Chakma et al. [34]
Acid Red B and Methylene Blue	AC, TiO_2_ and ZrFe_2_O_5_ based PC, and H_2_O_2_	Zirconium based catalyst was found to be more effective than TiO_2_ for AOPs, sono-photo-Fenton was found to utilize photo and Fenton activity of ZrFe_2_O_5_	Chakma and Moholkar [35]
Acid Red B and Methylene Blue	AC, ZnO and Fe-doped ZnO PC	Doping of Fe improves visible light activity of ZnO, *for the case of* *ultrasound + UV + saturated reaction media*: 1.25 times increase in degradation after 60-minutes for Methylene Blue dye, comparable degradation for Acid Red B	Chakma and Moholkar [36]
Triazhophos	HC, Fenton and Ozonation	Ozonation coupled with HC gave highest TOC removal (96%), ozonation introduced in the holding tank more effective than introduction in orifice	Gogate and Patil [37]
Acid Blue 80	H_2_O_2_, ozonation, Potassium persulfate, TiO_2_ and Fe-doped TiO_2_ PC	Loading of 0.4 g/L Potassium persulfate with optimized catalyst loading (0.2 g/L) and UV parameters resulted in > 99% degradation of dye	Ambati and Gogate [38]

Numerous papers on cavitation-based coupling of AOPs report parametric studies and binary/ternary combinations for pollutant degradation. They lack a truly ‘sensible’ approach for coupling AOPs and unnecessarily complicate the process to achieve higher removal efficiency. The complicated hybrid AOPs also lack a fundamental understanding of the importance and role of each AOP for degradation. With current knowledge in phenol degradation, it is evident that photocatalysis is a better AOP amongst others for mediating its oxidation. In this study, we optimized ZnO-mediated photocatalytic oxidation for phenol degradation, followed by sensible complementary approaches involving cavitation-mediated pre-treatment of ZnO or a cavitation-photocatalysis-peroxide based hybrid AOP. The hybrid approach investigated pulsed AC, a novel staggered H_2_O_2_ addition and hydrodynamic cavitation (HC) as hybrid options for maximising phenol oxidation. A novel phenomenon known as the ‘*pseudo staggered effect*’ was also observed and established in HC mediated photocatalysis-peroxide hybrid process for the first time.

Hybrid AOPs for pollutant degradation should prioritize the most influential AOP, based on the pollutant's nature, followed by a complementary AOP to enhance oxidation. This strategy is lacking in current literature. Thus, this study aims to fill this gap by investigating whether cavitation truly complements photocatalysis for phenol degradation by performing the following:

(i) The shear intensity (cavitational bubble collapse) was harnessed to pre-treat the photocatalyst
(ii) Optimised photocatalytic (PC) conditions were first determined for the hybrid process
(iii) Following this, PC was coupled with AC under optimised conditions for improved oxidation
(iv) Staggered addition of peroxide was investigated here as an additional novel strategy
(v) Finally, the AC-PC hybrid was translated to a HC-PC based process for phenol oxidation for the first time

The proposed strategies present several ways for researchers to approach hybrid AOPs for pollutant oxidation from wastewaters. Designing a ‘sensible’ approach based on these strategies yields a meaningful, reliable, targeted, and scalable wastewater treatment method.

## 2 Materials and methods

### 2.1 Materials

Phenol (>=99%, CAS number: 108–95-2), zinc oxide (CAS number: 1314–13-2) hydrogen peroxide (30%), hydrochloric acid (37%), sodium hydroxide pellets, and acetic acid (>=99%) were procured from Sigma Aldrich. HPLC grade Acetonitrile (99.6%) was procured from Fischer Scientific-UK. Ultra-pure water was produced on-site by a Milli-Q system with a specific resistivity of 18.2 MΩ.cm at 25 °C. UV-LED light strips (Model: SMD3528, Wavelength: 370 nm, Power specifications: 48 W) were procured from Amazon UK. A water pump with 0.5 HP capacity (Model: PKm60, Brand: Pedrollo Italy) was used for the HC experiments. Plastic pipes and pipe fittings were procured from a local vendor in Jeddah city, KSA.

### 2.2 Experimental methodology

A stock solution of phenol with concentration of 10,000 ppm was prepared by dissolving 10 g of phenol in 1 L of DI water. The stock solution was subsequently diluted with DI water to the required initial concentrations of 50 ppm for PC, acoustic cavitation – photocatalysis (AC – PC) and hydrodynamic cavitation – photocatalysis (HC – PC) experiments. For the experiments where hydrogen peroxide (H_2_O_2_) was added as an additional source of oxidant, the concentration of dosing is explicitly mentioned and this was either added once after the initial sample (sample at time, t = 0 min) or in a staggered manner at times (t = 0, 5 and 15 min).

#### 2.2.1 Choice of catalyst

ZnO powder was used as a photocatalyst in this study and was directly used as bought or subjected to sonication pre-treatments in either neutral (unaltered), acidic (pH 3) or basic (pH 10) solutions. Sonication pre-treatments (amplitude = 90% and duty cycle = 50% [1 s ON – 1 s OFF] with a Hielscher UP400 system) were carried out by taking 10 g of catalyst in 400 g of DI water, for durations of 15 and 30 min. Desired pH was adjusted with either 5 M HCl or 5 M NaOH. Upon pre-treatment, the catalyst was recovered from these solutions via centrifugation in a Thermo-Fischer Sorvall™ Legend™ XF, operated at 10,000 RPM for 15 min. The centrifuged catalyst was then dried overnight in an oven at 120 °C to evaporate any residual moisture. The dried powder was then used for characterization and for further experiments.

#### 2.2.2 Photocatalytic experiments

**Fig. 1:**
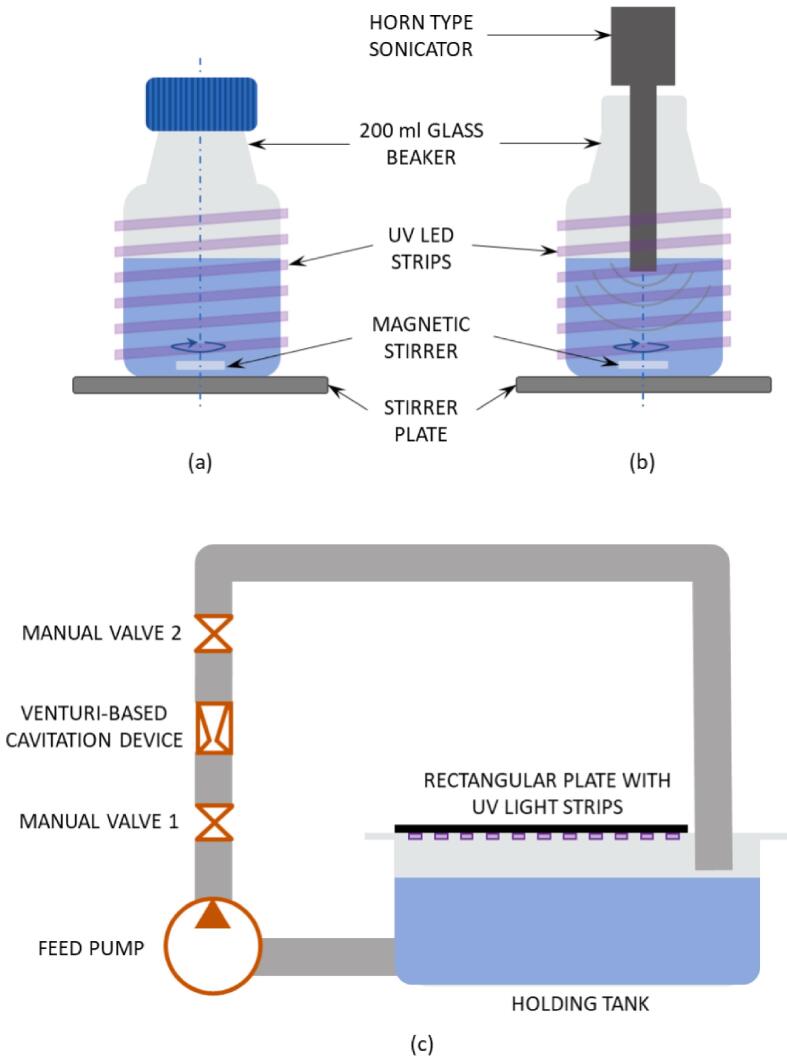
Schematic of experimental set-ups used: (a) Photocatalytic (PC) treatment, (b) AC + PC and (c) HC + PC. Experiments were carried out at an initial phenol concentration, C_0_ = 50 ppm phenol.

PC experiments were carried out in a 200 mL beaker containing an aqueous solution of phenol and ZnO catalyst as shown in Fig. 1a. Light cages were designed in a similar manner to that of a previous study by Pang et al. [39] as shown in Figure S1. The intensity of the light source was measured with a COHERENT FieldMaxxII-TOP light energy meter. The instrument was zeroed, and the wavelength was set at 370 nm. The light intensity at the bottom of beaker was 55 ± 2.5 W/cm^2^ at 370 nm (see Figure S1). The surface area (the light intensity measured in the axial direction was 175 ± 8 W/cm^2^ at 370 nm) of these UV light strips on the cylindrical metallic wire are similar to the one subsequently described in Section 2.2.4.

For the experiments performed on the beaker scale (photocatalytic: Section 2.2.2 and acoustic cavitation – photocatalysis: Section 2.2.3), the UV light passes through borosilicate glass and a ∼10% reduction in light intensity due to filtering is expected for a 370 nm source [40]. In a typical PC experiment, 1 mL of the phenol stock solution was added to 199 mL of DI water and mixed, to obtain a 50 ppm phenol solution. To this solution, a pre-determined quantity of photocatalyst was added and mixed for two minutes. Subsequently, a sample was collected, and this was marked as ‘sample prior to dark adsorption period’. The dark adsorption period was determined separately by performing experiments in the dark (with catalyst, without light). Similarly, a ‘light control’ experiment was performed with light but without catalyst. The loss of phenol during the dark conditions was attributed to adsorption to the catalyst surface and any loss of phenol due to light was subtracted from the net removal (negligible loss was observed – consistent with literature [41], [42] and hence no subtraction was necessary). After the dark adsorption period (typically 15 min), the UV light was switched on and samples were collected at dedicated time intervals. The initial dark adsorption experiment to determine adsorption–desorption equilibrium was performed for 30 min (although the equilibrium was achieved in 10 min), the adsorption of phenol on the photocatalyst was found to be 18 – 20% (profile for dark adsorption is presented in Figure S2), while the loss of phenol due to UV light was found to be negligible. The collected samples were centrifuged at 15,000 rpm for 15 min to separate the catalyst. The supernatant was collected into a HPLC vial for analysis. Experiments were performed in duplicates unless otherwise specified and the errors quantified were ± 4%.

#### 2.2.3 Acoustic cavitation – Photocatalysis experiments

The AC-PC experimental setup was similar manner to that used for PC experiments except that there was a sonication probe immersed into the liquid (shown in Fig. 1b and Figure S3). A Hielscher UP400 probe was used for acoustic cavitation, the conditions used for introducing cavitation were 90% amplitude and 50% duty cycle [1 s ON – 1 s OFF] for all experimental conditions. The procedure for sample collection was similar to that used for the PC experiments.

#### 2.2.4 Hydrodynamic cavitation – Photocatalysis experiments

HC experiments were performed on a rig (as depicted in Fig. 1c) similar to that used in previous studies [43], [44]. A venturi, as described in the work of Simpson and Ranade [45], was used as the HC device. The throat diameter (d_t_) of the device was 4 mm and the inception window for this device was 50 – 55 kPag [44]. The operating pressure drop was 100 kPag (flow rate of 14.5 LPM), the temperature was maintained at 20 ± 2 °C for all the experiments and the working volume was 3.5 L. The HC loop employed a Grundfos PKm60 pump (pump curve presented in Figure S4), the light intensity inside the HC holding tank was measured with the COHERENT FieldMaxxII-TOP light energy meter was 175 ± 8 W/cm^2^ at 370 nm (shown on Figure S5).

In the case of HC-PC experiments, the UV light source is not shielded by any surface (unlike the glass beaker, in the case of PC and AC-PC experiments). However, the light source introduced into the HC-PC tank is illuminated to a tank containing 10–12 cm of catalyst-liquid slurry. Despite ensuring a consistency in similar surface area of light strips, these unintended/minor light intensity differences exist in the two (AC-PC and HC-PC) techniques in this study.

### 2.3 High performance liquid chromatography (HPLC)

Phenol concentration was analysed by Agilent HPLC, using a UV detector, a C18 column (5 µm, 150 × 4.6 m ID), thermostat maintained at 40 °C. The mobile phase used was 35.2% Acetonitrile and 64.8% DI water (v/v basis). The mobile phase flowrate was set to 1 mL/min and the method run time was 8 min. A calibration curve was prepared with the peak area obtained at 270 nm.

### 2.4 Characterization

In a Micromeritics ASAP 2040, N_2_ physisorption (-196 °C) was performed with a priori sample, degassing for 10 h at 250 °C. The BET surface area was computed using the Brunauer–Emmet–Teller (BET) equation from the resulting isotherms. High-resolution XPS analyses were performed in a Kratos Axis Ultra DLD spectrometer. Equipped with a monochromatic Al Kα x-ray source (hν = 1486.6 eV) that operates at 150 W and a multichannel plate and delay line detector under a vacuum of 1 ∼ 10^-9^ mbar, spectra were collected at energies of 160 and 20 eV, respectively. All samples were loaded in floating mode to avoid differential charging before charge neutralization. Reported binding energies were referenced to the C 1 s peak of the (C-C, C-H) bond, set at 285.0 eV. CasaXPS software was used for processing the spectra. UV–Vis spectra for each of the catalytic samples was collected within the light wavelength of 200–800 nm on a Jasco V670 UV–Vis-NIR spectrophotometer. Prior to each measurement, the instrument was calibrated using barium sulphate as a standard.

## 3 Results and discussions

**Fig. 2:**
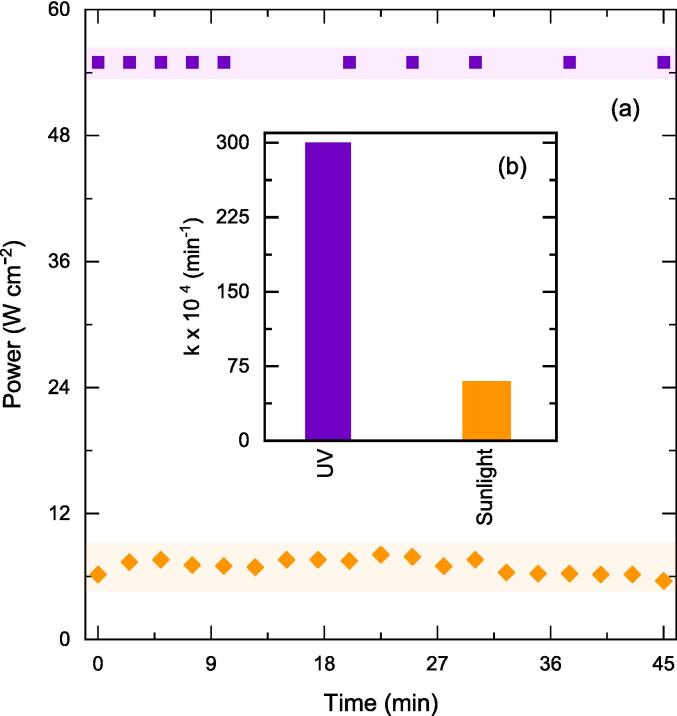
(a) Light intensity for respective illuminations at λ = 370 nm and (b) Influence of illumination type on the removal of phenol. Other operating conditions: V = 200 mL, C_0_ = 50 ppm, C_cat_ = 0.5 g/L, unchanged pH.

In this work, commercial as received ZnO (0.5 g/L) was taken as such, and its phenol degradation performance was investigated under artificial (UV) and natural (solar) illumination. The optimization of relevant PC parameters such as catalyst loading and aqueous phase pH for conversion of phenol was investigated in our recent study [46]. The first investigation was to test the possibility of using natural solar light. In Fig. 2 (a), the light intensity measured at 370 nm (minimum wavelength required for excitation of ZnO) through the duration of the experiment for both solar and ultraviolet light is plotted. Light is the driving force for photocatalytic reactions and the intensity of 370 nm from sunlight was about seven times lower than that of UV light. This is expected because, <5% of the solar spectrum is composed of UV light with majority of it in the UV-A region (315–400 nm)[47].

The resulting rate constant for conversion of phenol shows a nearly five-fold increase in the case of UV light as compared to natural solar light and is plotted as Fig. 2 (b). Therefore, UV radiation was used as a light source for all the laboratory-scale experiments in this work. However, the exploitation of natural solar light for organic molecules-laden wastewater treatment does deserve a more detailed study for regions (like Kingdom of Saudi Arabia and rest of the middle east) where sunlight is abundant for a major portion of the year. The exploitation of natural light for wastewater treatment would thereby mean lesser resource utilization.

### 3.1 Catalyst pre-treatment

The first parameter considered in this work that affects a photocatalytic system for phenol oxidation was the catalyst pre-treatment. Sonication was selected as a pre-treatment method for the ZnO catalyst. The basis to investigate this route was to identify if sonication had a beneficial effect by increasing the specific surface area, to enable higher surface mediated oxidation. For this purpose, a fixed amount of catalyst was suspended in water and subjected to sonication in a beaker under the influence of ultrasound horn for 15 min and 30 min duration. The catalysts were then recovered and dried as mentioned previously in section 2.2.1 and then used for further experiments.

#### 3.1.1 Pre-treatment in unaltered pH

**Fig. 3:**
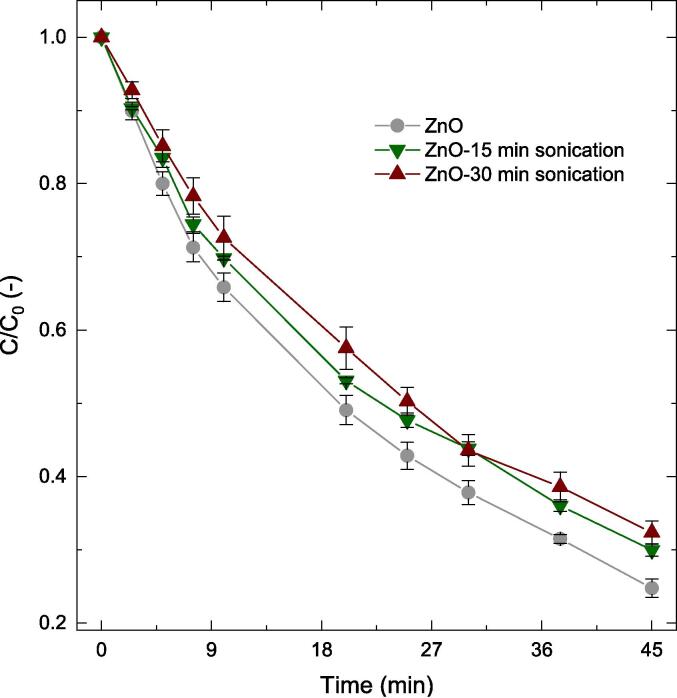
Influence of catalyst pre-treatment on the removal of phenol. Other operating conditions: V = 200 mL, C_0_ = 50 ppm, C_cat_ = 0.5 g/L, unchanged pH.

As can be seen in Fig. 3, the effect of ZnO pre-treatment in water with unchanged pH had only a marginal effect on phenol removal compared to the purely photocatalytic route. It is likely that the surface charge of the catalyst was unvaried through sonication, leading to its performance similar to that of the untreated catalyst. Photocatalyst agglomeration upon sonication may have been a possibility leading to a slightly lower oxidation performance. BET characterization were performed on the three catalysts to indicate if this was the case. BET revealed the catalyst surface area decreased from 6 m^2^ g^−1^ for the as received ZnO to 4–5 m^2^ g^−1^ for both the 15 min and 30 min sonicated ZnO in unchanged pH. Similar observation has been reported previously where, beyond a threshold specific cavitation energy input, re-agglomeration of metal oxides can occur [48], [49]. Particle agglomeration under sonication may be due to enhanced particle–particle interactions. This could be a result of the increased collision frequency and a favourable reduction in free energy as reported by Taurozzi et al. [49] While this behaviour upon sonication is undesired, it is not uncommon. Since the scope of this work was to identify a potential route of catalyst pre-treatment as a hybrid route (amongst other investigated hybrids) and not examine this in any further depth, no additional analysis was performed. It is however suggested that FESEM or DLS techniques could be exploited to explore this and confirm this behaviour.

#### 3.1.2 Pre-treatment in acidic and basic pH

**Fig. 4:**
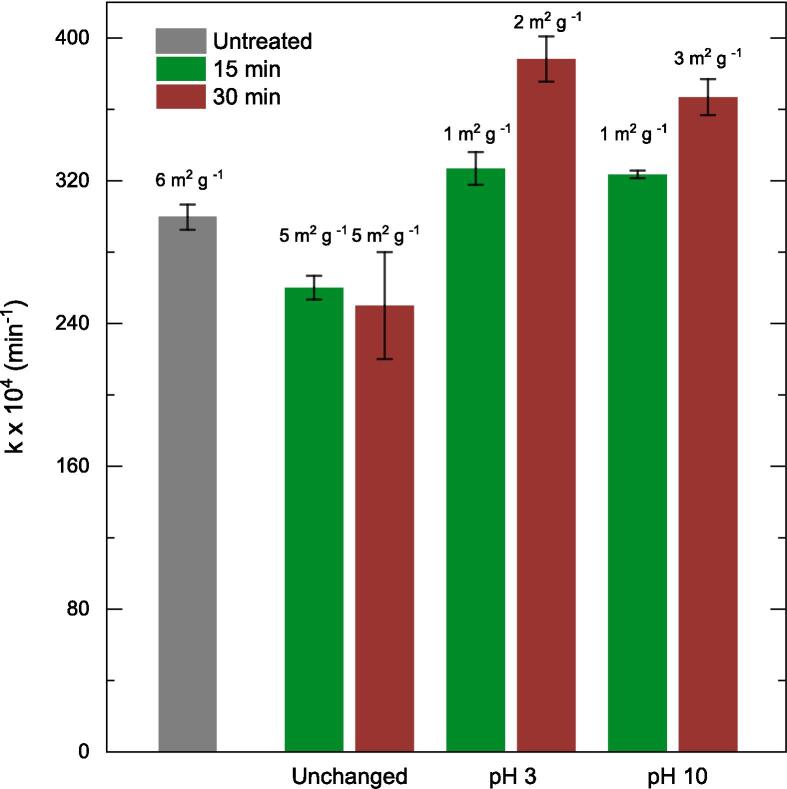
Influence of pH and sonication time on the catalyst pre-treatment on the pseudo-first order rate constant. Values atop the bars refer to the respective BET-measured surface areas. Other operating conditions: V = 200 mL, C_0_ = 50 ppm, C_cat_ = 0.5 g/L, pH = 3–10.

The solution pH has an effect on the catalyst performance through modifying the surface charge [50] and influencing the positions of conduction-valence bands [51]. In order to explore these options further, sonication treatment of the catalyst was performed at pH 3 and 10 for both 15 and 30 min. The surface area of the treated catalysts under modified pH also exhibited a similar trend to the treated catalysts in an unchanged pH environment. Fig. 4 validated that the activity of the catalyst is irrelevant as compared to the surface area available. Rather, is controlled by other factors; one of them being the pH of the pre-treatment medium.

In order to further discriminate between the catalysts and understand the activity through characterization, UV–Vis spectroscopy measurements were performed and reported in Figure S6. Across the light wavelengths tested, all the catalysts showed a similar absorption spectrum and little inferences could be drawn about the changes to their band positions.

**Fig. 5:**
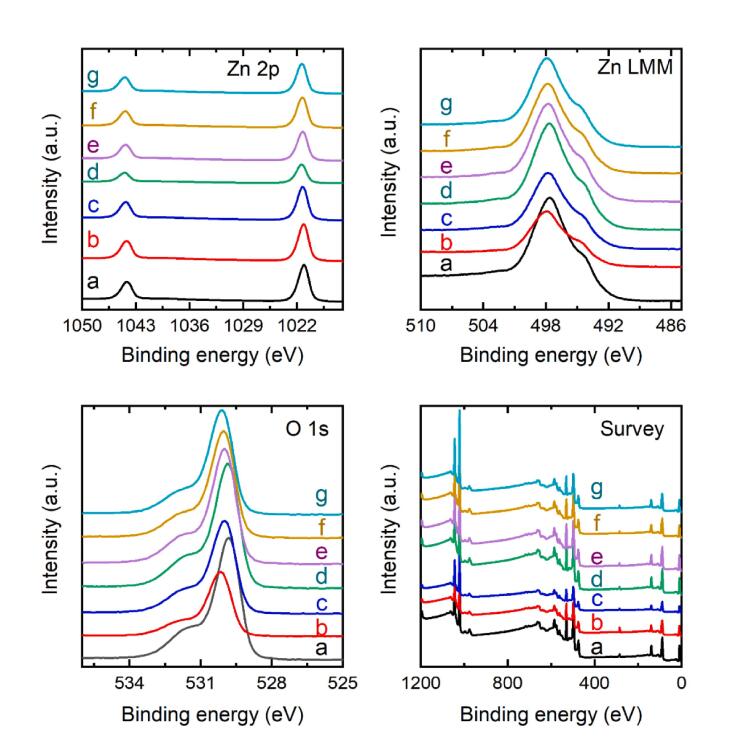
XPS spectra of all the ZnO photocatalysts before and after sonication at different pH and time intervals. a) ZnO-untreated, ZnO-sonicated at pH = 3 for b) 15 min and c) 30 min. ZnO-sonicated at pH = unchanged for d) 15 min and e) 30 min. ZnO-sonicated at pH = 10 for f) 15 min and g) 30 min.

XPS analysis was employed to collect the surface chemical states of these involved ZnO samples, and their corresponding oxygen environment (Fig. 5). Firstly, the presence of all elements (Zn, and O) were confirmed by collecting the survey spectrum of all the samples. As shown in Fig. 5, the O 1 s spectra of samples are asymmetric and consistently fitted by two distinct peaks (at 530.1 eV and 531.6 eV), which were noted as O_α_, and O_β_ respectively. The Oα and Oβ peak corresponding to the lattice oxygen and surface oxygen vacancies of ZnO [52]. For the Zn 2p spectrum, two peaks at ∼1021 eV and ∼1044 eV could be observed that could be assigned to the Zn 2p_3/2_ and Zn 2p_1/2_ states, respectively [53]. These results demonstrate that Zn existed in the samples with a divalent oxidation state in all the samples with no significant differences and is further confirmed by the Zn LMM spectra of these samples.

The Point of Zero Charge (PZC) of ZnO is ∼9 i.e. ZnO surface is positively charged when pH < 9 and negatively charged otherwise. Solution pH influences the ionisation state of ZnO surface, thereby controlling the photocatalytic behaviour. However under acidic conditions, phenol remains mainly in the neutral form and can be adsorbed on the catalyst surface, resulting in its oxidation via the active surface species [54]. This mechanism is absent in the alkaline range where repulsion between like charges of the phenol and ZnO is much greater [55].

Although many such studies have correlated the catalytic phenol decomposition with the solution pH and the PZC of ZnO; often studies have also shown a lack of such observation [56]. Anju et al. [21], recently reported a decrease of phenol conversion at pH 5.5; much before the PZC at pH 9 and attributed the same to the size and nature of particle dispersion and the type of catalyst. Thus, the need of diverging from a simple surface charge model is highlighted while addressing the positive effect of alkaline pre-treatment conditions as reported in this work [57], [58]. Such a study, if complemented with the reaction mixture analysis in terms of phenol oxidation products would further deepen the understanding the relation between photocatalysis and pre-treatment.

Further results discussed in the subsequent sections are therefore exclusively based on the as received photocatalysts. The results of PC based phenol oxidation and its enhancement mediated via AC and HC alongside peroxide addition sheds more meaningful light for both understanding the fundamentals as well as exploiting the approach for real world treatment scenarios.

### 3.2 Coupling photocatalysis with acoustic cavitation (AC)

**Fig. 6:**
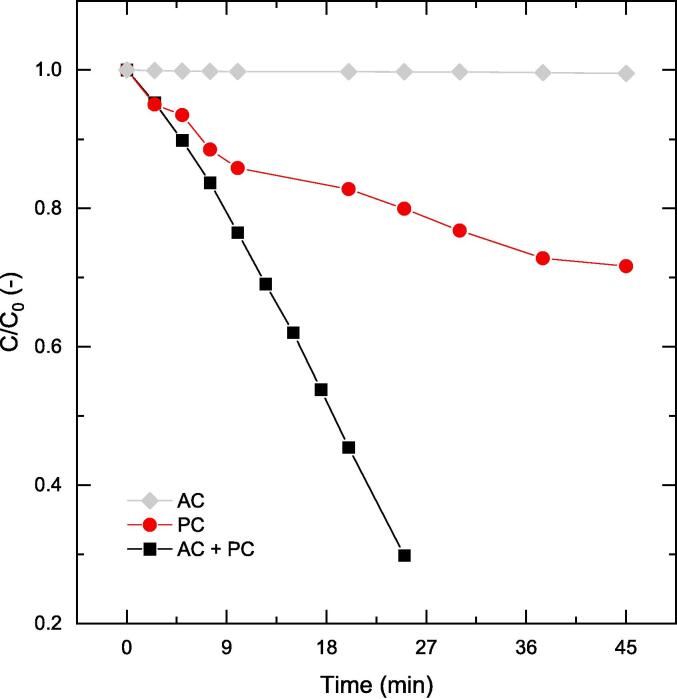
Influence of PC-only (stirring) vs PC + AC; both with 600 ppm hydrogen peroxide loading on the removal of phenol. Other operating conditions: V = 200 mL, C_0_ = 50 ppm, C_cat_ = 0.5 g/L, unchanged pH.

The next process choice comes from the combination of PC (as received ZnO) with AC. AC has the potential to intensify PC processes multiple fold as compared to standalone PC and harnessing the synergy of these two processes has already been shown in the literature [11]. Fig. 6 compares the phenol oxidation data for two modes: standalone – PC (i.e. by stirring) and PC + AC, both performed in the presence of hydrogen peroxide. Up to 70% of phenol degradation can be achieved by combining AC with PC.

As seen from Fig. 6, the phenol oxidation profiles due to PC suggests formation of intermediates that may have interfered with the adsorption of phenol on to the ZnO surface. The intermediates may have a higher affinity to the catalyst surface compared to phenol and would have resulted in an oxidation profile that was composed of multiple stages. For instance, with the operating pH of ∼7, the surface of ZnO would be positively charged (PZC ∼ 9.0[59]) whereas, with phenol (pKa ∼ 10.0[60]) being a weak acid, it would be in its protonated form. Therefore, the repulsion of like charges would have led to a lower adsorption of phenol on the catalyst. When an intermediate of higher affinity (pKa < 10) is formed, adsorption of this intermediate would have been favoured while hindering the oxidation of phenol. Since the intermediates were not analysed in this work, it is difficult to comment on what specific compounds may have been produced as intermediates in this oxidation process.

AC on its own without the addition of hydrogen peroxide was unable to oxidise phenol (Fig. 6) which is consistent with the data available in current literature [26]. This is due to the fact that polar compounds tend to exist in the bulk of the liquid (as they do not enter the cavitation bubble or reach the vicinity of the bubble–liquid interface) and do not interact with OH• created by the cavitation process [26]. The polarity of phenol does not support its diffusion to the bubble–liquid interface or into the bubble. The oxidation of phenol in such an instance is entirely dependent on the diffusion of oxidising radical species to the bulk. This is however unlikely as highly oxidising radicals such as the OH• have lifetimes that are shorter than the timelines required for diffusion [61].

In contrast to both the observed cases, the AC + PC + H_2_O_2_ process showed a smooth phenol oxidation profile with an increased rate of oxidation. This could be attributed to a number of reasons. For instance, as in the case of a PC + H_2_O_2_ system, intermediates might have formed. However, the complementary association of AC would have enabled the surface cleansing of the catalyst to allow phenol re-adsorption. Furthermore, if the intermediates produced are more hydrophobic, their diffusion through to the bubble–liquid interface or the cavitation bubble core would have improved their chances of degradation during AC to improve the overall performance of phenol oxidation. The UV light source employed does not result in the dissociation of H_2_O_2_[62], thus, incorporation of AC improved the phenol oxidation performance in a PC + H_2_O_2_ system.

### 3.3 Various modes of hydrogen peroxide addition

Another common route recommended in the literature for the PC-based process enhancement follows addition of oxidants like hydrogen peroxide (and ozone). H_2_O_2_ acts as a donor for OH•, which in turn react with the pollutant molecules and accelerate their oxidation. So, as a next logical step, hydrogen peroxide was used as an oxidant along with as received ZnO as a photocatalyst.

#### 3.3.1 PC + H_2_O_2_

**Fig. 7:**
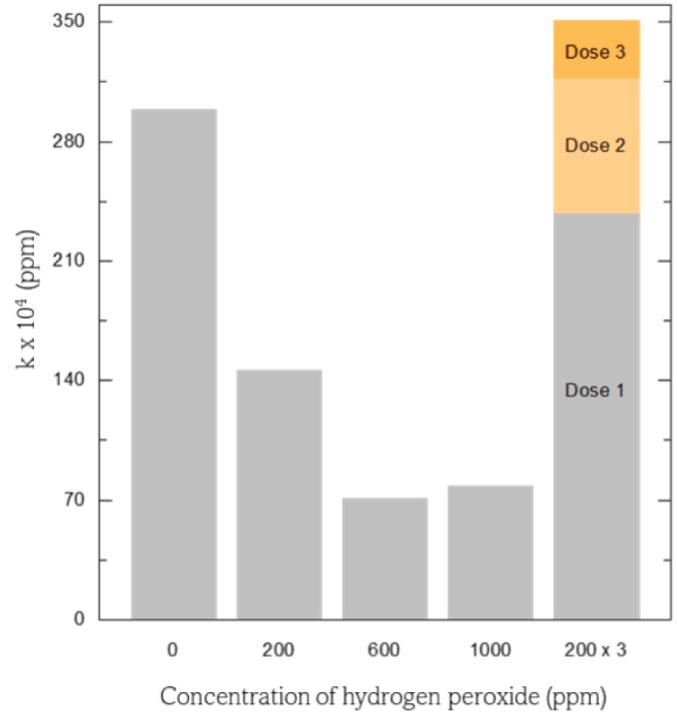
Influence of hydrogen peroxide loading on the removal of phenol*.* Other operating conditions: V = 200 mL, C_0_ = 50 ppm, C_cat_ = 0.5 g/L, pH = unchanged.

For this study, at t = 0 min, 50 ppm phenol, 200 mL water and 0–1000 ppm (0, 200, 600 and 1000) hydrogen peroxide was exposed to UV light for a period of 45 min. Fig. 7 shows a negative effect of peroxide addition on phenol oxidation during photocatalysis. Higher the peroxide dosing, lower the phenol removal and thereby lower the first order rate constant for the process. It is likely that the phenol (pKa = 10.0) and peroxide (pKa = 11.6) were in its protonated form in solution at a pH of ∼7.0. With the ZnO particles exhibiting a positive surface charge, the repulsion of peroxide molecules would have led to its minimal adsorption. Additionally, a possible competing adsorption between phenol and increasing peroxide concentration for the catalyst surface would have also led to a decreasing oxidation performance. Therefore, hydrogen peroxide addition for process improvement needs to be carefully investigated within the context of phenol conversion [63]. To further study the effect of peroxide dosing, a novel approach of staggered addition of peroxide was considered for the 600 ppm concentration that showed the least phenol oxidation.

Contrasting to a single addition of 600 ppm H_2_O_2_ at the start of the experiment, if the 600 ppm H_2_O_2_ was dosed in 3 batches of 200 ppm each (Fig. 7, last column), the process showed an enhanced oxidation performance. Staggered peroxide addition led to replenishing the H_2_O_2_ throughout the reaction as it proceeded rather than letting it decompose in the highly oxidising environment as with the single addition case. In the case of a single addition of 600 ppm, H_2_O_2_ was in stoichiometric excess when compared to phenol, therefore, peroxide dissociation via PC was favoured with possible loss due to water formation which is consistent with literature [63]. Lower concentration of H_2_O_2_ will induce a less likely competition between phenol and H_2_O_2_ adsorption on the catalyst surface (e.g., single addition of 200 ppm vs 600 ppm peroxide) and hence the staggered addition of smaller peroxide concentrations seemed to be a sensible approach. While we show that staggered addition of peroxide (and possibly other additives) may be a beneficial and thus a ‘sensible’ strategy to drive oxidation via hybrid processes (primarily PC driven), 200 ppm X 3 by no means is the optimised dosing. The demonstration of such a concept was to identify staggered dosing as a sensible approach. It is therefore recommended that each hybrid process utilising peroxide is optimised for staggered dosing as required using the demonstrated approach.

#### 3.3.2 PC + AC + H_2_O_2_

**Fig. 8:**
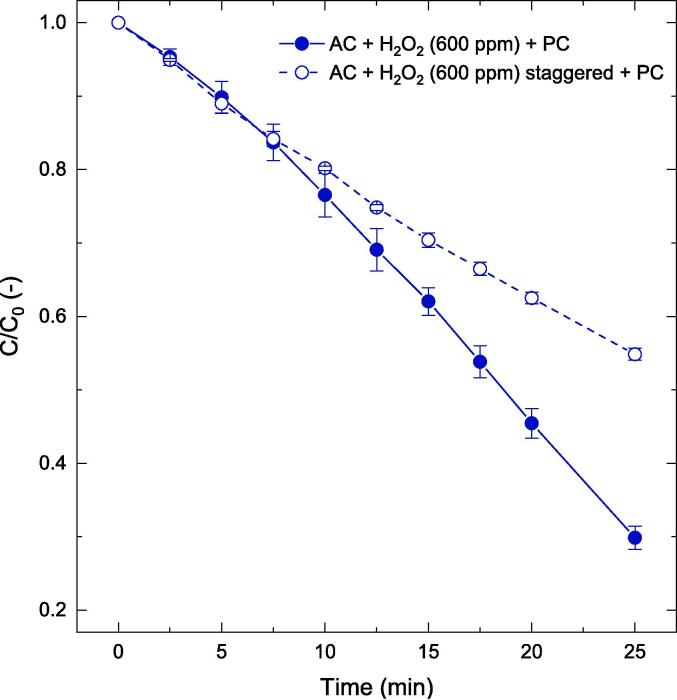
Influence of AC on the PC process – for the removal of phenol for staggered and one-step peroxide addition. Other operating conditions: V = 200 mL, C_0_ = 50-ppm, C_cat_ = 0.5 g/L, unchanged pH.

Further extending the findings from the previous section 3.3.1 to a PC + AC based hybrid process, staggered (200 ppm X 3) vs one time (600 ppm) addition of peroxide was investigated for phenol oxidation (Fig. 8). It appears that for a coupled AC-PC system, the staggered dosing was less beneficial compared to the one-time peroxide addition. The additional hydrodynamic effects introduced by the horn immersed in the reactor would probably result in improper oxidant utilization.

In order to quantify the effects observed with staggered peroxide addition, we introduce the enhancement factor (η), which can be defined as follows.

$$\eta =\frac{\mathrm{phenol}\;removal\;by\;staggered\;dosing}{\mathrm{phenol}\;removal\;by\;one-step\;dosing}\text{(-)}$$

**Fig. 9:**
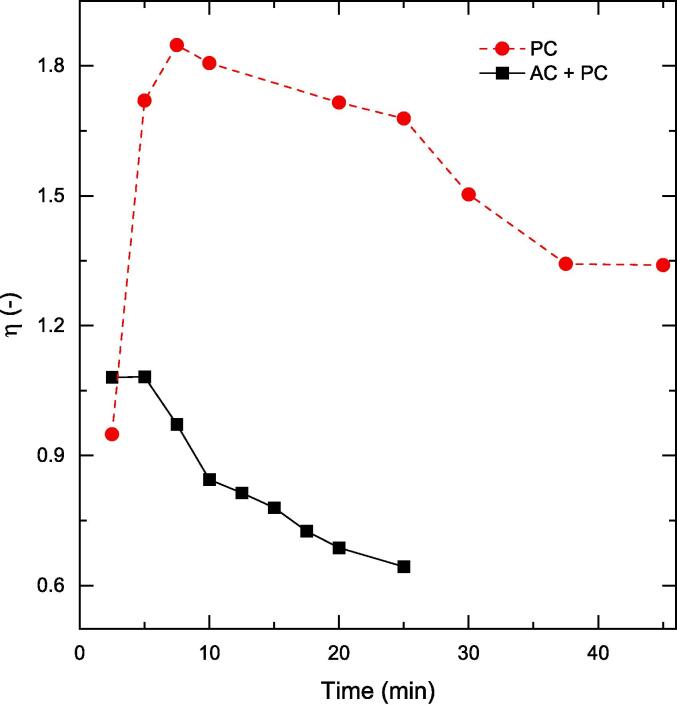
Effectiveness factor comparison for PC-only and a coupled PC-AC system. Other operating conditions: V = 200 mL, C_0_ = 50 ppm, C_cat_ = 0.5 g/L, unchanged pH.

Fig. 9 reveals that for PC, the first H_2_O_2_ dose led to rapid initial phenol oxidation. This is the simplest possible case where the reaction mixture had the catalyst, phenol and peroxide and hence the phenol oxidation was not influenced by factors such as oxidation intermediates but only the competition for phenol and peroxide for the catalyst surface. With staggered addition, the second and the third dose of peroxide led to a declining phenol oxidation performance. With subsequent doses, the system complexity had significantly increased with the presence of oxidation intermediates. As mentioned in section 3.2, if the oxidation intermediates have a lower pKa compared to phenol (e.g., catechol or resorcinol), the competition for catalyst surface adsorption is more complex than a two-component system containing only phenol and peroxide. Therefore, while the staggered peroxide addition seemed to have a net enhancement effect with PC, the initial oxidation performance after first peroxide dosing had a dominant effect.

In the case of the AC-PC system, η was found to be ∼1 for the initial stages of the reaction (up to t = 5 min) but continued to decay thereafter. This suggests that cavitation might have accelerated the consumption or decomposition of the dosed H_2_O_2_ either through dissociation under the influence of action of cavitation or on the photocatalyst surface, thereby reducing the interaction of target pollutant and peroxide (or mediated OH•). Although, staggering seems a good strategy for the PC system, it does not augment the AC-PC system.

### 3.4 Coupling photocatalysis with hydrodynamic cavitation (HC)

**Fig. 10:**
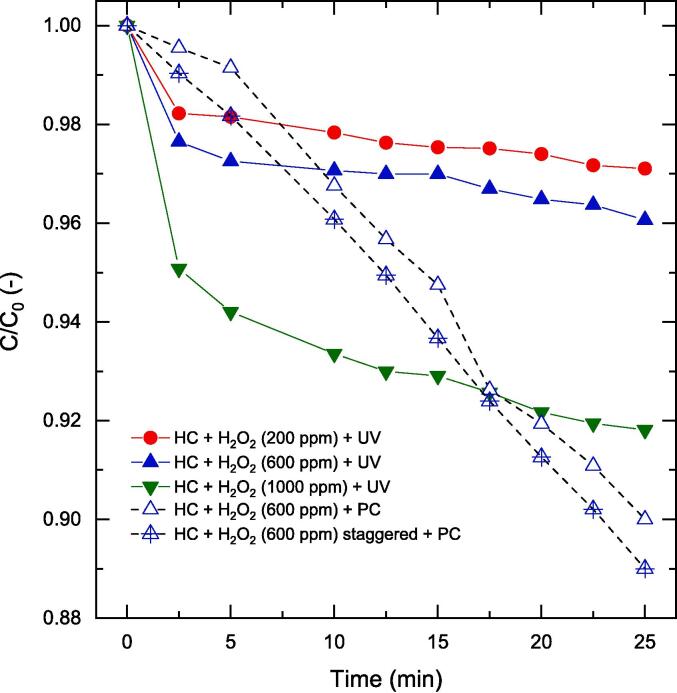
Effect of staggered and one-time addition of H_2_O_2_ for HC-PC system for phenol oxidation. Other operating conditions: V = 200 mL, C_0_ = 50 ppm, C_cat_ = 0.5 g/L, unchanged pH.

Unlike AC, HC is scalable and is therefore of increased relevance to wastewater treatment industries. In this work, with a venturi-based HC device and using the approach identified and validated hitherto, we investigated a combined HC-PC process with staggered and single addition of H_2_O_2_ (Fig. 10). Firstly, we investigated the phenol oxidation performance via HC with three different single peroxide doses (200 ppm, 600 ppm and 1000 ppm) in the presence of UV light - the UV light source employed does not result in the dissociation of H_2_O_2_[62]. Contrasting to PC and H_2_O_2_ mediated phenol oxidation results reported in Fig. 7, the HC based process (HC + H_2_O_2_) saw an increase in phenol oxidation performance with increase in peroxide concentration. While H_2_O_2_ was added to the holding tank at the beginning of the experiment, only a fraction of the peroxide enters the ‘active cavitation zone’ downstream of the venturi throat. This means that a major fraction of the excess peroxide in the system would still be available for oxidation throughout the process. We term this phenomenon as the ‘*pseudo staggered effect*’. Despite this new phenomenon devised here, the rate of peroxide utilisation towards phenol oxidation may differ as system complexity increases with the increasing production of oxidation intermediates. This is clearly observed in all the three cases where the initial rate of phenol oxidation (t < 5 min) is rapid compared to the rest of the process.

To overcome the issue with decaying degradation rate, PC based processes have been extremely beneficial due to factors such as surface mediated direct and indirect oxidation and oxidation in the bulk via diffused oxidising species. This was evident from our results in Fig. 3 with PC and peroxide based oxidation enhanced via synergistic AC. We therefore anticipated a similar trend in phenol oxidation performance as shown in Fig. 10.

With 600 ppm peroxide addition to the HC system in the presence of PC, the decaying oxidation rates beyond 5 min was eliminated. This is because of the higher interaction of oxidation intermediates with the photocatalyst leading to their degradation. The combined degradation of phenol and its oxidation intermediates was therefore possible. With staggered addition of peroxide, a similar trend with only a slightly higher rate was observed. This validates the case that PC based oxidation can be enhanced via systematic integration of hybrid processes. The lack of significant difference in oxidation performance with and without staggered peroxide addition can be explained by the ‘*pseudo staggered effect*’ as the excess peroxide present in the system was utilised on demand and could be a function of the ‘active cavitation zone’ volume. The addition of oxidants such as ozone at the throat of a cavitation device have been demonstrated elsewhere [64], [65], [66] and offers a potential case where the excess peroxide might not have to be dosed at the beginning of the experiment. This would also mean that the ‘pseudo staggered effect’ observed otherwise could be overcome.

### 3.5 HC vs AC for coupling with PC

While comparing the phenol concentrations at the end of HC-PC with AC-PC (with peroxide in both cases), it may appear that AC is the preferred mode of cavitation for coupling with PC. However, a closer look at the data reveals a different story. Firstly, the scales of operation are far apart. AC-PC occurs on a beaker-scale of 200 mL; while the HC device works with a holding tank of 4 L with a working volume of 3.5 L, being continuously fed by a pump at 14–15 LPM to work in a recirculating batch mode. Under these conditions, the overall phenol conversion accounts to ∼10% from HC-PC as compared to ∼70% (best case). This translates into 20 mg phenol removed per reactor volume for HC-PC, which is higher than 7 mg for the AC-PC case, identifying a higher net phenol removal from HC in same time. Secondly, the CAPEX-OPEX costs associated with AC are significantly higher comparted to HC, which become even more significant as the scale rises. To further compare the HC-PC and AC-PC systems, cavitational yield calculations were performed. For the same extent of removal (10%), phenol removed per unit power consumed (mg J^−1^) was determined for the HC-PC and AC-PC systems (as tabulated in Table S1). The relevant quantities such as volume, time for 10% removal, power dissipated [67], and mg of phenol were tabulated. Based on this calculation, the cavitational yields for phenol oxidation via HC-PC and AC-PC were found to be 4.83 × 10^-4^ and 9.09 × 10^-5^ mg J^−1^ respectively. The translates to the HC-PC process being >5 times more efficient (with a smaller specific energy input) than its AC-PC counterpart for phenol oxidation. Considering all these factors, HC-PC can offer additional enhancements to phenol oxidation at large operating scales.

## 4 Conclusions

Phenol being a polar compound is typically recalcitrant to its oxidation when treated with standalone (scalable) cavitation-based processes. However, photocatalytic removal of phenol though potentially favourable, has slower oxidation kinetics due to the intermediates formed in the process interfering with the parent phenol oxidation. To overcome these limitations, process intensification of photocatalytic phenol removal via systematic integration of cavitation and hydrogen peroxide was performed in this work. While a range of hybrid advanced oxidation processes have been reported in literature for removing phenols, none of them have managed to address the phenol oxidation systematically.

A photocatalyst pre-treatment route via sonication prior to phenol oxidation was first investigated. Compared to the performance of the as received ZnO photocatalyst, 30 min sonicated ZnO at pH 3 improved the phenol oxidation rate by ∼25%. To understand whether addition of hydrogen peroxide improved the phenol oxidation rates, three different peroxide concentrations in stoichiometric excess (200 ppm, 600 ppm and 1000 ppm) were added to the photocatalysis system. The rate of phenol oxidation decreased by >50% (200 ppm) and >70% (600 ppm and 1000 ppm) with the addition of peroxide. Instead of a single addition of excess peroxide, a staggered addition mode was introduced in this work for the first time. This meant adding 200 ppm peroxide 3 times over the experimental timeframe to achieve a cumulative addition of 600 ppm. This resulted in >15% increase in the oxidation rates compared to as received photocatalysts.

Next, cavitation (AC) was coupled with the photocatalysis-peroxide system for phenol oxidation. It was determined that staggered addition did not positively influence the hybrid process, whereas the single addition of 600 ppm peroxide lead to >70% removal of phenol in 25 min. By defining the enhancement factor (η), it was established that the initial rate of oxidation was faster for the aforementioned hybrid process mainly due to the less complex reaction mixture (two component system composed of phenol and peroxide). With increase in reaction time, the phenol oxidation intermediates resulted in a complex reaction mixture that led to a decline in oxidation rates due to competitive surface adsorption on the catalysts.

HC coupled with the photocatalyst-peroxide system on the other hand exhibited an increased oxidation rate and phenol removal with increase in peroxide concentration. We define this phenomenon for the first time as the ‘*pseudo staggered effect*’, where the stoichiometric excess peroxide in the system would still be available for oxidation throughout the process as only a small fraction of peroxide is present in the ‘active cavitation zone (HC only)’ at any given time during the process. If the ‘*pseudo staggered effect*’ were to be true, a proper staggered addition of peroxide should have minimal influence on the oxidation rates. This was indeed true and established strongly in this work paving way for HC based photocatalytic hybrid pollutant treatment systems. From an industrial perspective, in terms of phenol removal per system volume, this translates to 7 mg L^-1^ vs 20 mg L^-1^ for AC and HC based hybrid photocatalytic systems respectively. The cavitational yields were also superior for HC based systems and enabled >5 times more phenol oxidation compared to its AC based counterpart. We have thus demonstrated that it is indeed possible to ‘sensibly’ combine cavitation and photocatalysis unlike other non-systematic existing literature-based approaches for an effective hybrid AOP. The nature of the pollutant under investigation must be the driver in these cases for designing ‘sensible’ hybrid AOPs.

## CRediT authorship contribution statement

**Varaha P. Sarvothaman:** Conceptualization, Methodology, Validation, Writing – original draft. **Vijay K. Velisoju:** Validation. **Janardhanraj Subburaj:** Methodology. **Mebin S. Panithasan:** Validation. **Shekhar R. Kulkarni:** Validation, Data curation. **Pedro Castaño:** Supervision. **James Turner:** Supervision. **Paolo Guida:** Writing – review & editing, Supervision. **William L. Roberts:** Funding acquisition, Supervision, Resources. **Sanjay Nagarajan:** Conceptualization, Methodology, Writing – review & editing, Visualization, Supervision.

## Declaration of Competing Interest

The authors declare that they have no known competing financial interests or personal relationships that could have appeared to influence the work reported in this paper.

## Data Availability

The authors are unable or have chosen not to specify which data has been used.

## References

[1] Ghafoori S., Omar M., Koutahzadeh N., Zendehboudi S., Malhas R.N., Mohamed M., Al-Zubaidi S., Redha K., Baraki F., Mehrvar M. (2022). New advancements, challenges, and future needs on treatment of oilfield produced water: A state-of-the-art review. Sep. Purif. Technol..

[2] Sun Y., Liu Y.i., Chen J., Huang Y., Lu H., Yuan W., Yang Q., Hu J., Yu B., Wang D., Xu W., Wang H. (2021). Physical pretreatment of petroleum refinery wastewater instead of chemicals addition for collaborative removal of oil and suspended solids. J. Clean. Prod..

[3] Qiao X., Zhang Z., Yu J., Ye X. (2008). Performance characteristics of a hybrid membrane pilot-scale plant for oilfield-produced wastewater. Desalination.

[4] Crini G., Lichtfouse E. (2019). Advantages and disadvantages of techniques used for wastewater treatment. Environ. Chem. Lett..

[5] Elmobarak W.F., Hameed B.H., Almomani F., Abdullah A.Z. (2021). A review on the treatment of petroleum refinery wastewater using advanced oxidation processes. Catalysts.

[6] Villegas L.G.C., Mashhadi N., Chen M., Mukherjee D., Taylor K.E., Biswas N. (2016). A short review of techniques for phenol removal from wastewater. Current Pollution Reports.

[7] Nosaka Y., Nosaka A. (2016). Understanding hydroxyl radical (• OH) generation processes in photocatalysis. ACS Energy Lett..

[8] Chen Y., Yang S., Wang K., Lou L. (2005). Role of primary active species and TiO2 surface characteristic in UV-illuminated photodegradation of Acid Orange 7. J. Photochem. Photobiol. A Chem..

[9] Sundar K.P., Kanmani S. (2020). Progression of Photocatalytic reactors and it’s comparison: A Review. Chem. Eng. Res. Des..

[10] Thanekar P., Gogate P. (2018). Application of hydrodynamic cavitation reactors for treatment of wastewater containing organic pollutants: intensification using hybrid approaches. Fluids.

[11] Gogate P.R., Pandit A.B. (2004). Sonophotocatalytic reactors for wastewater treatment: a critical review. AIChE J.

[12] Gogate P.R., Mujumdar S., Pandit A.B. (2002). A Sonophotochemical Reactor for the Removal of Formic Acid from Wastewater. Ind. Eng. Chem. Res..

[13] Asli S.A., Taghizadeh M. (2020). Sonophotocatalytic Degradation of Pollutants by ZnO-Based Catalysts: A Review. ChemistrySelect.

[14] Ragaini V., Selli E., Letizia Bianchi C., Pirola C. (2001). Sono-photocatalytic degradation of 2-chlorophenol in water: kinetic and energetic comparison with other techniques. Ultrason. Sonochem..

[15] Maezawa A., Nakadoi H., Suzuki K., Furusawa T., Suzuki Y., Uchida S. (2007). Treatment of dye wastewater by using photo-catalytic oxidation with sonication. Ultrason. Sonochem..

[16] Panchangam S.C., Lin A.Y., Tsai J.H., Lin C.F. (2009). Sonication-assisted photocatalytic decomposition of perfluorooctanoic acid. Chemosphere.

[17] Torres R.A., Nieto J.I., Combet E., Pétrier C., Pulgarin C. (2008). Influence of TiO2 concentration on the synergistic effect between photocatalysis and high-frequency ultrasound for organic pollutant mineralization in water. Appl Catal B.

[18] Bethi B., Sonawane S.H., Rohit G.S., Holkar C.R., Pinjari D.V., Bhanvase B.A., Pandit A.B. (2016). Investigation of TiO2 photocatalyst performance for decolorization in the presence of hydrodynamic cavitation as hybrid AOP. Ultrason. Sonochem..

[19] Wang X., Jia J., Wang Y. (2017). Combination of photocatalysis with hydrodynamic cavitation for degradation of tetracycline. Chem. Eng. J..

[20] Bagal M.V., Gogate P.R. (2014). Degradation of diclofenac sodium using combined processes based on hydrodynamic cavitation and heterogeneous photocatalysis. Ultrason. Sonochem..

[21] Anju S.G., Yesodharan S., Yesodharan E.P. (2012). Zinc oxide mediated sonophotocatalytic degradation of phenol in water. Chem. Eng. J..

[22] Wang X., Jia J., Wang Y. (2011). Degradation of CI Reactive Red 2 through photocatalysis coupled with water jet cavitation. J. Hazard. Mater..

[23] Suslick K.S., Mdleleni M.M., Ries J.T. (1997). Chemistry Induced by Hydrodynamic Cavitation. J. Am. Chem. Soc..

[24] Destaillats H., Hung H.-M., Hoffmann M.R. (2000). Degradation of Alkylphenol Ethoxylate Surfactants in Water with Ultrasonic Irradiation. Environ. Sci. Tech..

[25] Ranade V.V., Bhandari V.M., Nagarajan S., Sarvothaman V.P., Simpson A.T. (2022).

[26] Berlan J., Trabelsi F., Delmas H., Wilhelm A.M., Petrignani J.F. (1994). Oxidative degradation of phenol in aqueous media using ultrasound. Ultrason. Sonochem..

[27] Wu C., Liu X., Wei D., Fan J., Wang L. (2001). Photosonochemical degradation of phenol in water. Water Res..

[28] Uddin M.H., Hayashi S. (2009). Effects of dissolved gases and pH on sonolysis of 2, 4-dichlorophenol. J. Hazard. Mater..

[29] Ahmed S., Rasul M.G., Martens W.N., Brown R., Hashib M.A. (2010). Heterogeneous photocatalytic degradation of phenols in wastewater: a review on current status and developments. Desalination.

[30] Gaya U.I., Abdullah A.H. (2008). Heterogeneous photocatalytic degradation of organic contaminants over titanium dioxide: A review of fundamentals, progress and problems. J. Photochem. Photobiol. C: Photochem. Rev..

[31] Ani I.J., Akpan U.G., Olutoye M.A., Hameed B.H. (2018). Photocatalytic degradation of pollutants in petroleum refinery wastewater by TiO2- and ZnO-based photocatalysts: Recent development. J. Clean. Prod..

[32] Dinesh G.K., Pramod M., Chakma S. (2020). Sonochemical synthesis of amphoteric Cu0-Nanoparticles using Hibiscus rosa-sinensis extract and their applications for degradation of 5-fluorouracil and lovastatin drugs. J. Hazard. Mater..

[33] Kumar M.S., Sonawane S.H., Pandit A.B. (2017). Degradation of methylene blue dye in aqueous solution using hydrodynamic cavitation based hybrid advanced oxidation processes. Chem. Eng. Process..

[34] Chakma S., Praneeth S., Moholkar V.S. (2017). Mechanistic investigations in sono-hybrid (ultrasound/Fe2+/UVC) techniques of persulfate activation for degradation of Azorubine. Ultrason. Sonochem..

[35] Chakma S., Moholkar V.S. (2015). Sonochemical synthesis of mesoporous ZrFe 2 O 5 and its application for degradation of recalcitrant pollutants. RSC Adv..

[36] Chakma S., Moholkar V.S. (2015). Investigation in mechanistic issues of sonocatalysis and sonophotocatalysis using pure and doped photocatalysts. Ultrason. Sonochem..

[37] Gogate P.R., Patil P.N. (2015). Combined treatment technology based on synergism between hydrodynamic cavitation and advanced oxidation processes. Ultrason. Sonochem..

[38] Ambati R., Gogate P.R. (2017). Photocatalytic degradation of Acid Blue 80 using iron doped TiO2 catalyst: Understanding the effect of operating parameters and combinations for synergism. J. Water Process Eng..

[39] Pang X., Sarvothaman V.P., Skillen N., Wang Z., Rooney D.W., Ranade V.V., Robertson P.K.J. (2022). Kinetic modelling of the photocatalytic degradation of Diisobutyl phthalate and coupling with acoustic cavitation. Chem. Eng. J..

[40] McMurray T., Byrne J., Dunlop P., McAdams E. (2005). Photocatalytic and electrochemically assisted photocatalytic oxidation of formic acid on TiO 2 films under UVA and UVB irradiation. J. Appl. Electrochem..

[41] Chiou C.-H., Wu C.-Y., Juang R.-S. (2008). Influence of operating parameters on photocatalytic degradation of phenol in UV/TiO2 process. Chem. Eng. J..

[42] Vaiano V., Matarangolo M., Murcia J.J., Rojas H., Navío J.A., Hidalgo M.C. (2018). Enhanced photocatalytic removal of phenol from aqueous solutions using ZnO modified with Ag. Appl Catal B.

[43] Rajoriya S., Bargole S., Saharan V.K. (2017). Degradation of a cationic dye (Rhodamine 6G) using hydrodynamic cavitation coupled with other oxidative agents: Reaction mechanism and pathway. Ultrason. Sonochem..

[44] Sarvothaman V.P., Simpson A., Ranade V.V. (2020). Comparison of Hydrodynamic Cavitation Devices Based on Linear and Swirling Flows: Degradation of Dichloroaniline in Water. Ind. Eng. Chem. Res..

[45] Simpson A., Ranade V.V. (2019). 110th Anniversary: Comparison of Cavitation Devices Based on Linear and Swirling Flows: Hydrodynamic Characteristics. Ind. Eng. Chem. Res..

[46] Sarvothaman, V. P., Subburaj, J., Velisoju, V.K, Kulkarni, S.R., Nagarajan, S., Castaño, P., Farooq, A., Roberts, W.L., Degradation of phenol by a hybrid oxidation approach:systematic coupling of suspended photocatalysis with hydrodynamic cavitation. Submitted to Industrial Engineering & Chemistry (Research). ed.; 2023.

[47] Smoke T., Smoking I. (2004). IARC monographs on the evaluation of carcinogenic risks to humans. IARC, Lyon.

[48] Thonglerth P., Sujaridworakun P., Boondamnoen O. (2022). Preparation of ZnO Nanoparticles Water-based Dispersion. J. Phys.: Conf. Ser..

[49] Taurozzi J.S., Hackley V.A., Wiesner M.R. (2011). Ultrasonic dispersion of nanoparticles for environmental, health and safety assessment–issues and recommendations. Nanotoxicology.

[50] Haque M.M., Muneer M., Bahnemann D.W. (2006). Semiconductor-Mediated Photocatalyzed Degradation of a Herbicide Derivative, Chlorotoluron, in Aqueous Suspensions. Environ. Sci. Technol..

[51] Chong M.N., Jin B., Chow C.W.K., Saint C. (2010). Recent developments in photocatalytic water treatment technology: A review. Water Res..

[52] Wang J., Xia Y., Dong Y., Chen R., Xiang L., Komarneni S. (2016). Defect-rich ZnO nanosheets of high surface area as an efficient visible-light photocatalyst. Appl. Catal. B.

[53] Gao S., Yang F.u., Song C., Cai Q., Wang R., Zhou S., Kong Y. (2020). Photocatalytic producing dihydroxybenzenes from phenol enabled by gathering oxygen vacancies in ultrathin porous ZnO nanosheets. Appl. Surf. Sci..

[54] Saien J., Soleymani A.R. (2007). Degradation and mineralization of Direct Blue 71 in a circulating upflow reactor by UV/TiO2 process and employing a new method in kinetic study. J. Hazard. Mater..

[55] Saien J., Delavari H., Solymani A.R. (2010). Sono-assisted photocatalytic degradation of styrene-acrylic acid copolymer in aqueous media with nano titania particles and kinetic studies. J. Hazard. Mater..

[56] Karunakaran C., Dhanalakshmi R. (2008). Semiconductor-catalyzed degradation of phenols with sunlight. Sol. Energy Mater. Sol. Cells.

[57] Dewidar H., Nosier S.A., El-Shazly A.H. (2018). Photocatalytic degradation of phenol solution using Zinc Oxide/UV. J. Chem. Health Saf..

[58] Lee K.M., Lai C.W., Ngai K.S., Juan J.C. (2016). Recent developments of zinc oxide based photocatalyst in water treatment technology: a review. Water Res..

[59] Sabouni R., Gomaa H. (2019). Photocatalytic degradation of pharmaceutical micro-pollutants using ZnO. Environ. Sci. Pollut. Res..

[60] Gross K.C., Seybold P.G. (2001). Substituent effects on the physical properties and pKa of phenol. Int. J. Quantum Chem.

[61] De-Nasri S.J., Nagarajan S., Robertson P.K.J., Ranade V.V. (2021). Quantification of hydroxyl radicals in photocatalysis and acoustic cavitation: Utility of coumarin as a chemical probe. Chem. Eng. J..

[62] Li X., Chen C., Zhao J. (2001). Mechanism of photodecomposition of H2O2 on TiO2 surfaces under visible light irradiation. Langmuir.

[63] Tseng D.-H., Juang L.-C., Huang H.-H. (2012). Effect of oxygen and hydrogen peroxide on the photocatalytic degradation of monochlorobenzene in aqueous suspension. Int. J. Photoenergy.

[64] Rajoriya S., Bargole S., George S., Saharan V.K. (2018). Treatment of textile dyeing industry effluent using hydrodynamic cavitation in combination with advanced oxidation reagents. J. Hazard. Mater..

[65] Gore M.M., Saharan V.K., Pinjari D.V., Chavan P.V., Pandit A.B. (2014). Degradation of reactive orange 4 dye using hydrodynamic cavitation based hybrid techniques. Ultrason. Sonochem..

[66] Rajoriya S., Bargole S., Saharan V.K. (2017). Degradation of reactive blue 13 using hydrodynamic cavitation: Effect of geometrical parameters and different oxidizing additives. Ultrason. Sonochem..

[67] No Y., Son Y. (2019). Effects of probe position of 20 kHz sonicator on sonochemical oxidation activity. Jpn. J. Appl. Phys..

