# Supplementary material for: Is cavitation a truly sensible choice for intensifying photocatalytic oxidation processes? – Implications on phenol degradation using ZnO photocatalysts
Source: Ultrason Sonochem. 2023 Aug 3;99:106548. doi: 10.1016/j.ultsonch.2023.106548 (PMC10433233; doi:10.1016/j.ultsonch.2023.106548)
Supplement: Supplementary data 1 [file mmc1.docx]

Is Cavitation a Truly Sensible Choice for Intensifying Photocatalytic Oxidation Processes? – Implications on Phenol Degradation using ZnO photocatalysts

Varaha P. Sarvothaman^1^, Vijay K. Velisoju^2^, Janardhanraj Subburaj^1^, Mebin S. Panithasan^1^,

Shekhar R. Kulkarni^2,*^, Pedro Castaño^2^, James Turner^1^, Paolo Guida^1^, William L. Roberts^1,*^

and Sanjay Nagarajan^3,4*^

*^1^King Abdullah University of Science and Technology (KAUST), Clean Combustion Research Center, Thuwal 23955-6900 Saudi Arabia.*

*^2^Multiscale Reaction Engineering (MuRE) Group, KAUST Catalysis Center (KCC), King Abdullah University of Science and Technology (KAUST),*

*Thuwal 23955-6900 Saudi Arabia.*

*^3^Department of Chemical Engineering, University of Bath, Claverton Down, Bath BA2 7AY, UK.*

*^4^Centre for Sustainable Energy Systems, University of Bath, Claverton Down, Bath BA2 7AY, UK.*

*Email: sn908@bath.ac.uk, william.roberts@kaust.edu.sa and shekhar.kulkarni@kaust.edu.sa

Supporting information:


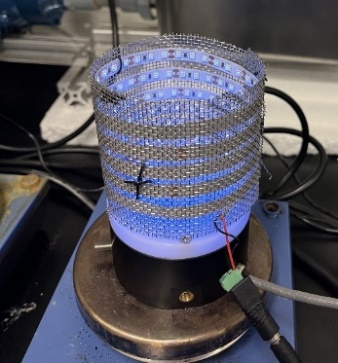

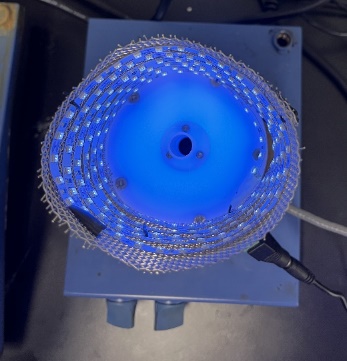


Figure S1. Photograph of UV light source and its intensity measurement with an energy meter for photocatalytic based experiments.


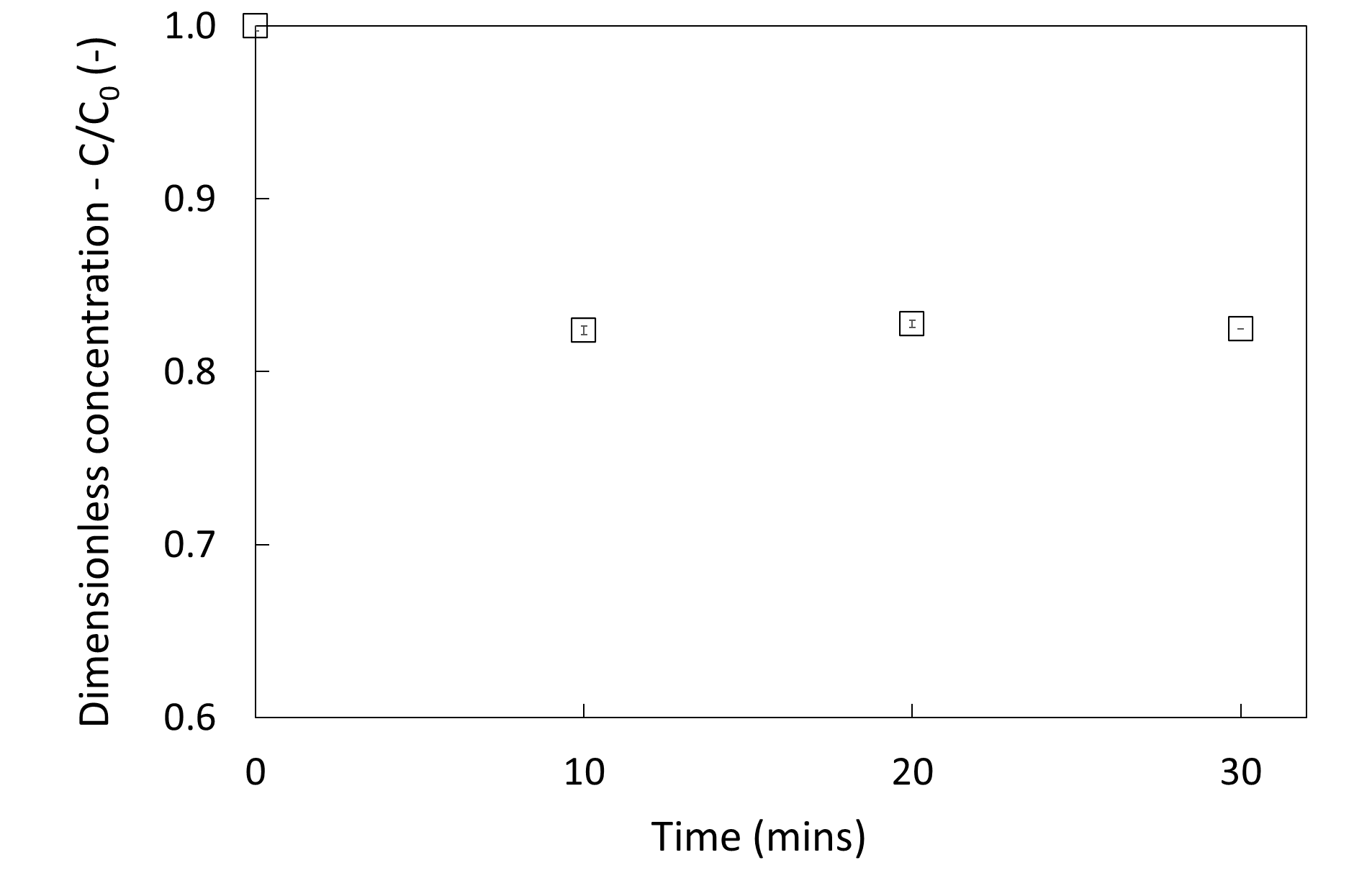


Figure S2. Concentration profile for dark adsorption of phenol prior to photocatalytic treatment: V = 200 mL, C_0_ = 50 ppm, C_cat_ = 0.5 g L-1, unchanged pH.


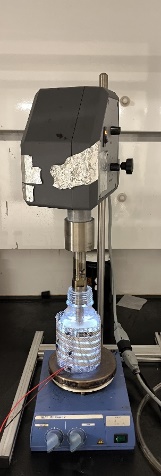


Figure S3. Photograph of experimental arrangement for AC – PC.


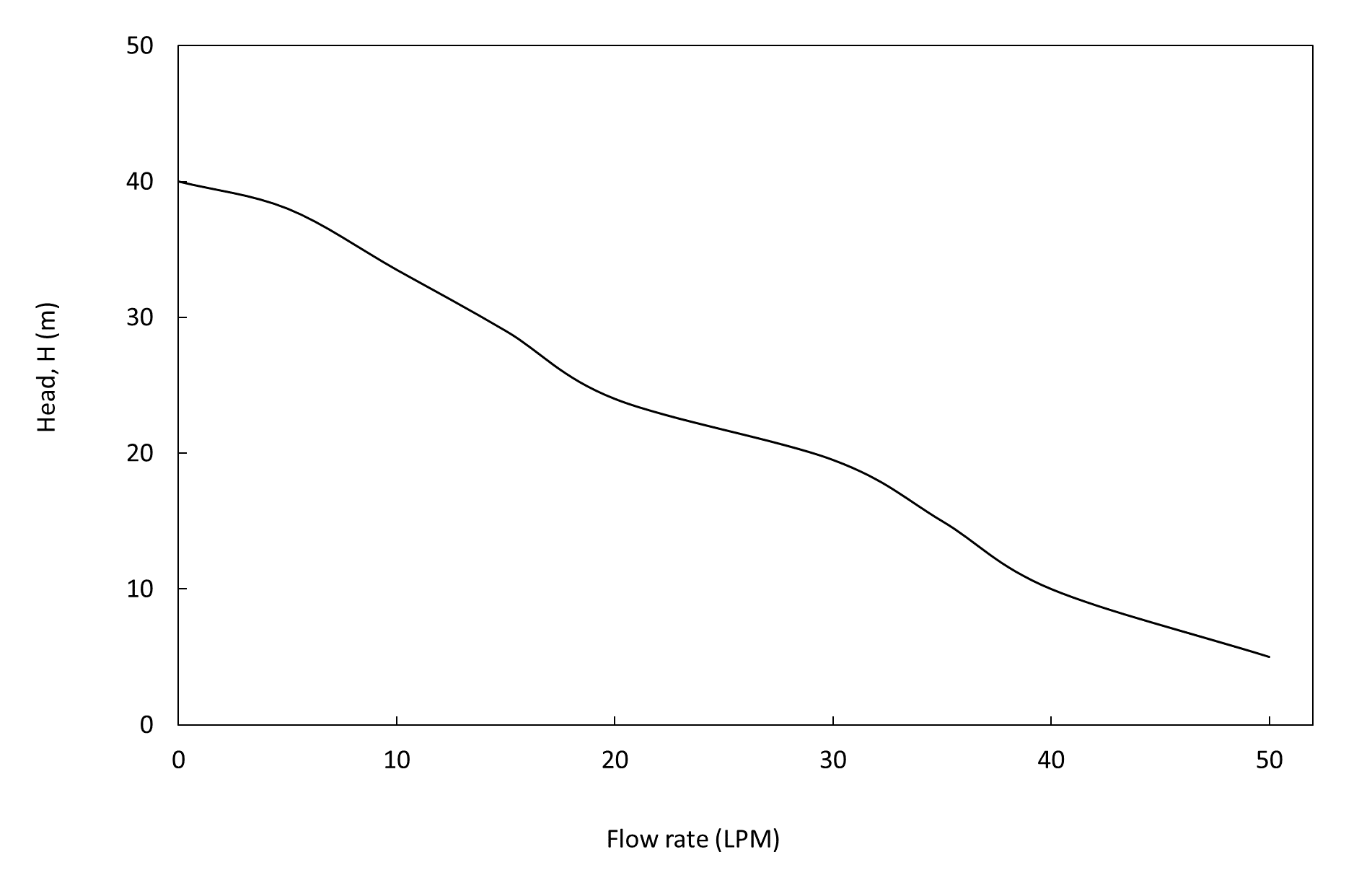


Figure S4. Pump curve for Grundfos Pkm 60.


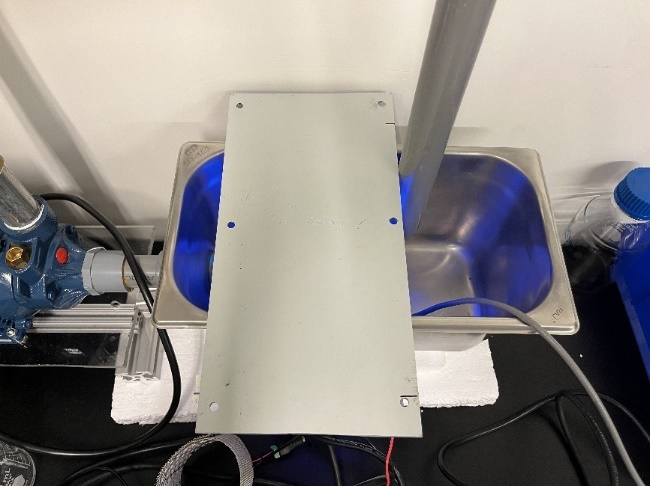


Figure S5. Photograph of placement of UV light source and its intensity measurement with a energy meter for HC – PC based experiments.

Figure S6. UV-Vis spectra for the catalysts pre-treated in varying pH solutions and sonication times.

Table S1. Calculation of energy efficiency of AC-PC and HC-PC processes.

|  | AC-PC | HC-PC |
| --- | --- | --- |
| Volume, V (mL) | 200 | 3500 |
| Time, t for 10% phenol oxidation (min) | 5.95 | 25 |
| Power dissipated for 10% reduction (J) | 10996 | 36250 |
| mg of phenol removed | 1.0 | 17.5 |
| phenol removed per unit power (mg/J) | 9.09E-05 | 4.83E-04 |
